# BROJA-2PID: A Robust Estimator for Bivariate Partial Information Decomposition

**DOI:** 10.3390/e20040271

**Published:** 2018-04-11

**Authors:** Abdullah Makkeh, Dirk Oliver Theis, Raul Vicente

**Affiliations:** Institute of Computer Science, University of Tartu, Ülikooli 17, 51014 Tartu, Estonia

**Keywords:** bivariate information decomposition, Cone Programming

## Abstract

Makkeh, Theis, and Vicente found that Cone Programming model is the most robust to compute the Bertschinger et al. partial information decomposition (BROJA PID) measure. We developed a production-quality robust software that computes the BROJA PID measure based on the Cone Programming model. In this paper, we prove the important property of strong duality for the Cone Program and prove an equivalence between the Cone Program and the original Convex problem. Then, we describe in detail our software, explain how to use it, and perform some experiments comparing it to other estimators. Finally, we show that the software can be extended to compute some quantities of a trivaraite PID measure.

## 1. Introduction

For random variables X,Y,Z with finite range, consider the mutual information MI(X;Y,Z): the amount of information that the pair (Y,Z) contain about *X*. How can we quantify the contributions of *Y* and *Z*, respectively, to MI(X;Y,Z)? This question is at the heart of *(bivariate) partial information decomposition (PID)* [1,2,3,4]. Information theorists agree that there can be: information shared redundantly by *Y* and *Z*; information contained uniquely within *Y* but not within *Z*; information contained uniquely within *Z* but not within *Y*; and information that synergistically results from combining both *Y* and *Z*. The quantities are denoted by: SI(X;Y,Z); UI(X;Y\Z), UI(X;Z\Y); and CI(X;Y,Z), respectively. All four of these quantities add up to MI(X;Y,Z); moreover, the quantity of total information that *Y* has about *X* is decomposed into the quantity of unique information that *Y* has about *X* and shared information that *Y* shares with *Z* about *X*, and similarly for quantity of total information that *Z* has about *X*, thus SI(X;Y,Z)+UI(X;Y\Z)=MI(X;Y), and SI(X;Y,Z)+UI(X;Z\Y)=MI(X;Z). Hence, if the joint distribution of (X,Y,Z) is known, then there is (at most) one degree of freedom in defining a bivariate PID. In other words, defining the value of one of the information quantities defines a bivariate PID.

Bertschinger et al. [1] have given a definition of a bivariate PID where the synergistic information is defined as follows:

(1) $$\mathrm{CI}\left(X;Y,Z\right):=\max(\mathrm{MI}\left(X;Y,Z\right)-\mathrm{MI}\left(X^{\prime };Y^{\prime },Z^{\prime }\right))$$

where the maximum extends over all triples of random variables $\left(X^{\prime },Y^{\prime },Z^{\prime }\right)$ with the same 12,13-marginals as (X,Y,Z), i.e., $P\left(X=x,Y=y\right)=P\left(X^{\prime }=x,Y^{\prime }=y\right)$ for all x,y and $P\left(X=x,Z=z\right)=P\left(X^{\prime }=x,Z^{\prime }=z\right)$ for all x,z. It can easily be verified that this amounts to maximizing a concave function over a compact, polyhedral set described by inequalities [5]. Hence, using standard theorems of convex optimization [5,6], BROJA’s bivariate PID can be efficiently approximated to any given precision.

In practice, computing CI has turned out to be quite challenging, owing to the fact that the objective function is not smooth on the boundary of the feasible region, which results in numerical difficulties for state-of-the-art interior point algorithms for solving convex optimization problems. We refer to [5] for a thorough discussion of this phenomenon.

Due to these challenges and the need in the scientific computing community to have a reliable easily usable software for computing the BROJA bivariate PID, we made available on GitHub a Python implementation of our best method for computing the BROJA bivariate PID (https://github.com/Abzinger/BROJA_2PID/). The solver is based on a conic formulation of the problem and thus a Cone Program is used to compute the BROJA bivariate PID. This paper has two contributions. Firstly, we prove the important property of strong duality for the Cone Program and prove an equivalence between the Cone Program and the original Convex problem (1). Secondly, we describe in detail our software and how to use it. Thirdly, we test the software against different instances and then compare the results with the computeUI estimator introduced in [7] and the ibroja estimator from the dit package (https://github.com/dit/dit). Finally, we show how to use the so-called exponential cone to model some quantities of the multivariate PID measure introduced in [8].

This paper is organized as follows. In the remainder of this section, we define some notation we will use throughout, and review the Convex Program for computing the BROJA bivariate PID from [1]. In the next section, we review the math underlying our software to the point which is necessary to understand how it works and how it is used. In Section 3, we walk the reader through an example of how to use the software, and then explain its inner workings and its use in detail. In Section 4, we present some computations on larger problem instances, discuss how the method scales up, and compare it to other methods. In Section 5, we present a modeling, using the exponential cone, of some quantities for a multivariate PID measure. We conclude the paper by discussing our plans for the future development of the code.

### Notation and Background

Denote by X the range of the random variable *X*, by Y the range of *Y*, and by Z the range of *Z*. We identify joint probability density functions with points in $R^{W}$; for example, the joint probability distribution of (X,Y,Z) is a vector in $R^{X\times Y\times Z}$. (We measure information in nats, unless otherwise stated.) We use the following notational convention.

An asterisk stands for “sum over everything that can be plugged in instead of the ∗”, e.g., if $p,q\in R^{X\times Y\times Z}$,

$$q_{x,y,*}=\sum_{w\in Z}q_{x,y,w};\;p_{*,y,z}q_{*,y,z}=\left(\sum_{u\in X}p_{u,y,z}\right)\left(\sum_{u\in X}q_{u,y,z}\right)$$

We do not use the symbol ∗ in any other context.

We define the following notation for the marginal distributions of (X,Y,Z): With *p* the joint probability density function of (X,Y,Z):

$$\begin{array}{cc} p_{x,y,*}=P\left(X=x\wedge Y=y\right) & \mathrm{for}\;\mathrm{all}\;x\in X,\;y\in Y\\ p_{x,*,z}=P\left(X=x\wedge Z=z\right) & \mathrm{for}\;\mathrm{all}\;x\in X,\;y\in Y. \end{array}$$

These notations allow us to write the Convex Program from [1] in a succinct way. Unraveling the objective function of (1), we find that, given the marginal conditions, it is equal, up to a constant not depending on $X^{\prime },Y^{\prime },Z^{\prime }$, to the conditional entropy $H(X^{\prime }|Y^{\prime },Z^{\prime })$. Replacing maximizing H(…) by minimizing −H(…), we find (1) to be equivalent to the following Convex Program:

(CP) $$\begin{array}{ccc} \mathrm{minimize}\; & \sum_{x,y,z}q_{x,y,z}\ln\frac{q_{x,y,z}}{q_{*,y,z}} & \mathrm{over}\;q\in R^{X\times Y\times Z}\\ \mathrm{subject}\;\mathrm{to}\;\; & q_{x,y,*}=p_{x,y,*} & \mathrm{for}\;\mathrm{all}\;(x,y)\in X\times Y\\ & q_{x,*,z}=p_{x,*,z} & \mathrm{for}\;\mathrm{all}\;(x,z)\in X\times Z\\ & q_{x,y,z}\geq 0 & \mathrm{for}\;\mathrm{all}\;(x,y,z)\in X\times Y\times Z. \end{array}$$

## 2. Cone Programming Model for Bivariate PID

In [5], we introduced a model for computing the BROJA bivariate PID based on a so-called “Cone Programming”. Cone Programming is a far reaching generalization of Linear Programming: The usual inequality constraints which occur in Linear Programs can be replaced by so-called “generalized inequalities”—see below for details. Similar to Linear Programs, dedicated software is available for Cone Programs, but each type of generalized inequalities (i.e., each cone) requires its own algorithms. The specific type of generalized inequalities needed for the computation of the BROJA bivariate PID requires solvers for the so-called “Exponential Cone”, of which two we are aware of ECOS [9] and SCS [10].

In the computational results of [5], we found that the Cone Programming approach (based on one of the available solvers) was, while not the fastest, the most robust of all methods for computing the BROJA bivariate PID which we tried, such as projected gradient descent, interior point for general convex programs, geometric programming, etc. The reason for this success is that the interior point method for Cone Programming is an extension of the efficient interior point methods of Linear Programming, see [11] for more details. This is why our software is based on the Exponential Cone Programming model.

In this section, we review the mathematical definitions to the point in which they are necessary to understand our model and the properties of the software based on it.

### 2.1. Background on Cone Programming

A nonempty *closed convex cone* $K\subseteq R^{m}$ is a closed set which is *convex*, i.e., for any x,y∈K and 0≤θ≤1 we have

θx+(1−θ)y∈K,

and is a *cone*, i.e., for any x∈K and θ≥0 we have

θx∈K;

for example, $R_{+}^{n}$ is a closed convex cone. *Cone Programming* is a far-reaching generalization of Linear Programming, which may contain so-called *generalized inequalities*: For a fixed closed convex cone $K\subseteq R^{m}$, the generalized inequality “$a\leq_{K}b$” denotes b−a∈K for any $a,b\in R^{m}$. Recall the primal-dual pair of Linear Programming. The *primal problem* is,

(2) $$\begin{array}{cc} \mathrm{minimize}\; & c^{T}w\\ \mathrm{subject}\;\mathrm{to}\; & Aw=b\\ & Gw\leq h \end{array}$$

over variable $w\in R^{n}$, where $A\in R^{m_{1}\times n},G\in R^{m_{2}\times n},c\in R^{n},b\in R^{m_{1}},$ and $h\in R^{m_{2}}$. Its *dual problem* is,

(3) $$\begin{array}{cc} \mathrm{maximize} & -b^{T}\eta -h^{T}\theta \\ \mathrm{subject}\;\mathrm{to} & -A^{T}\eta -G^{T}\theta =c\\ & \theta \geq 0. \end{array}$$

over variables $\eta \in R^{m_{1}}$ and $\theta \in R^{m_{2}}$. There are two properties that the pair (2) and (3) may or may not have, namely, weak and strong duality. The following defines the duality properties.

**Definition** **1.** *Consider a primal-dual pair of the Linear Program *(2)* and *(3)*. Then, we define the following,*

*1*. *A vector $w\in R^{n}$ (respectively, $\left(\eta ,\theta \right)\in R^{m_{1}}\times R^{m_{2}}$) is said to be a feasible solution of *(2)* (respectively, *(3)*) if Aw=b and $Gw\leq_{K}h$ (respectively, $-A^{T}\eta -G^{T}\theta =c$ and θ≥0), i.e., none of the constraints in *(2)* (respectively, *(3)*) are violated by w (respectively, (η,θ))*.
*2*. *We say that *(2)* and *(3)* satisfy weak duality if for all w and all (η,θ) feasible solutions of *(2)* and *(3)*, respectively,*
  $$-b^{T}\eta -h^{T}\theta \leq c^{T}w.$$
*3*. *If w is a feasible solution of *(2)* and (η,θ) is a feasible solution of *(3)*, then the duality gap d is*
  $$d:=c^{T}w+b^{T}\eta +h^{T}\theta .$$
*4*. *We say that *(2)* and *(3)* satisfy strong duality when the feasible solutions w and (η,θ) are optimal in *(2)* and *(3)*, respectively, if and only if d is zero*.

Weak duality always holds for a Linear Program, however strong duality holds for a Linear Program whenever a feasible solution of (2) or (3) exists. These duality properties are used to certify the optimality of *w* and (η,θ). The same concept of duality exists for Cone Programming, the *primal cone problem* is

(P) $$\begin{array}{cc} \mathrm{minimize} & c^{T}w\\ \mathrm{subject}\;\mathrm{to} & Aw=b\\ & Gw\leq_{K}h, \end{array}$$

over variable $w\in R^{n}$, where $A\in R^{m_{1}\times n},G\in R^{m_{2}\times n},c\in R^{n},b\in R^{m_{1}},$ and $h\in R^{m_{2}}$. The *dual cone problem* is,

(D) $$\begin{array}{cc} \mathrm{maximize} & -b^{T}\eta -h^{T}\theta \\ \mathrm{subject}\;\mathrm{to} & -A^{T}\eta -G^{T}\theta =c\\ & \theta \geq_{K^{*}}0, \end{array}$$

where $K^{*}:=\left\{u\in R^{n}|u^{T}v\geq 0\;\mathrm{for}\;\mathrm{all}\;v\in K\right\}$ is the *dual cone* of K. The entries of the vector $\eta \in R^{m_{1}}$ are called the *dual variables for equality constraints*, Aw=b. Those of $\theta \in R^{m_{2}}$ are the *dual variables for generalized inequalities*, $Gw\leq_{K}h$. The primal-dual pair of Conic Optimization (P) and (D) satisfies weak and strong duality in the same manner as the Linear Programming pair. In the following, we define the interior point of a Cone Program which is a necessary condition for strong duality (see Definition 1) to hold for the Conic Programming pair.

**Definition** **2.** *Consider a primal-dual pair of the Conic Optimization *(P)* and *(D)*. Then, the primal problem *(P)* has an interior point $\tilde{x}$ if,*

- *$\tilde{x}$ is a feasible solution of* (P).
- There exists ϵ>0 such that for any $y\in R^{n}$, we have y∈K whenever ${∥h-G\tilde{x}-y∥}_{2}\leq \epsilon .$

**Theorem** **1** (Theorem 4.7.1 [12]). *Consider a primal-dual pair of the Conic Optimization *(P)* and *(D)*. Let w and (η,θ) be the feasible solutions of *(P)* and *(D)*, respectively. Then,*

*1*. *Weak duality always hold for *(P)* and* (D).
*2*. *If $c^{T}w$ is finite and *(P)* has an interior point $\tilde{w}$, then strong duality holds for *(P)* and* (D).

If the requirements of Theorem 1 are met for a conic optimization problem, then weak and strong duality can be used as guarantees that the given solution of a Cone Program is optimal.

**Figure 1:**
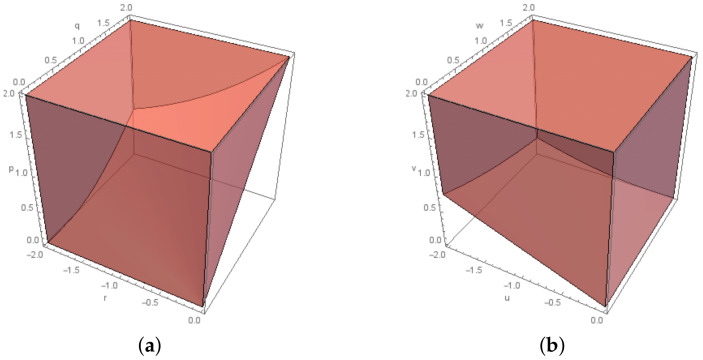
The $K_{\exp}$ cone and its dual: (**a**) $K_{\exp}$ for −2≤r≤0 and 0≤q,t≤2.; and (**b**) $K_{\exp}^{*}$ for −2≤u≤0 and 0≤w,v≤2.

One of the closed convex cones which we use throughout the paper is the *exponential cone*, $K_{\exp}$, (see Figure 1) defined in [13] as

(4) $$\left\{\left(r,t,q\right)\in R^{3}|q>0\;\mathrm{and}\;qe^{r/q}\leq t\right\}\cup \left\{\left(r,p,0\right)\in R^{3}|r\leq 0\;\mathrm{and}\;t\geq 0\right\},$$

which is the closure of the set

(5) $$\{\left(r,t,q\right)\in R^{3}|q>0\;\mathrm{and}\;qe^{r/q}\leq t\},$$

and its dual cone, $K_{\exp}^{*},$ (see Figure 1) is

(6) $$\left\{\left(u,v,w\right)\in R^{3}|u<0\;\mathrm{and}\;-u\cdot e^{w/u}\leq e\cdot v\right\}\cup \left\{\left(0,v,w\right)|v\geq 0\;\mathrm{and}\;w\geq 0\right\},$$

which is the closure of the set

(7) $$\{\left(u,v,w\right)\in R^{3}|u<0\;\mathrm{and}\;-u\cdot e^{w/u}\leq e\cdot v\}.$$

When $K=K_{\exp}$ in (P), the Cone Program is referred to as “Exponential Cone Program”.

### 2.2. The Exponential Cone Programming Model

The Convex Program (CP) which computes the bivariate partial information decomposition can be formulated as an Exponential Cone Program. Consider the following Exponential Cone Program where the variables are $r,t,q\in R^{X\times Y\times Z}$.

(EXP) $$\begin{array}{ccc} \mathrm{minimize} & -\sum_{x,y,z}r_{x,y,z}\\ \mathrm{subject}\;\mathrm{to}\; & q_{x,y,*}=p_{x,y,*} & \mathrm{for}\;\mathrm{all}\;(x,y)\in X\times Y\\ & q_{x,*,z}=p_{x,*,z} & \mathrm{for}\;\mathrm{all}\;(x,z)\in X\times Z\\ & q_{*,y,z}-t_{x,y,z}=0 & \mathrm{for}\;\mathrm{all}\;(x,y,z)\in X\times Y\times Z\\ & \left(-r_{x,y,z},-t_{x,y,z},-q_{x,y,z}\right)\leq_{K_{\exp}}0 & \mathrm{for}\;\mathrm{all}\;(x,y,z)\in X\times Y\times Z. \end{array}$$

The first two constraints are the *marginal equations* of (CP). The third constraints connects the *t*-variables with the *q*-variables which will be denoted as *coupling equations*. The generalized inequality connects these to the variables forming the objective function.

**Proposition** **1.** *The exponential cone program *(EXP)* is equivalent to the Convex Program* (CP).

**Proof.**  Let ${¶}_{\mathrm{CP}}\left(b\right)$ and ${¶}_{\exp}\left(b\right)$ be the feasible region of (CP) and (EXP), respectively. We define the following

(8) $$\begin{array}{ccc} f:{¶}_{\mathrm{CP}}\left(b\right) & \to & {¶}_{\exp}\left(b\right)\\ q_{x,y,z} & \to & f\left(q_{x,y,z}\right):=\left\{\begin{array}{cc} \left(q_{x,y,z}\ln\frac{q_{*,y,z}}{q_{x,y,z}},q_{*,y,z},q_{x,y,z}\right) & \mathrm{if}\;q_{xyz}>0\\ \left(0,q_{*,y,z},q_{x,y,z}\right) & \mathrm{if}\;q_{x,y,z}=0. \end{array}\right. \end{array}$$

For $q_{x,y,z}\in {¶}_{\mathrm{CP}}$, we have

$${\left(-1,0,0\right)}^{T}\cdot f\left(q_{x,y,z}\right)=\left\{\begin{array}{cc} q_{x,y,z}\ln\frac{q_{x,y,z}}{q_{*,y,z}} & \mathrm{if}\;q_{xyz}>0\\ 0 & \mathrm{if}\;q_{xyz}=0 \end{array}\right.$$

and since conditional entropy at $q_{x,y,z}=0$ vanishes, then the objective function of (CP) evaluated at $q\in {¶}_{\mathrm{CP}}$ is equal to that of (EXP) evaluated at f(q). If (r,t,q)∈¶exp\Im(f), then there exists x,y,z such that $r_{x,y,z}<q_{x,y,z}\ln\frac{t_{x,y,z}}{q_{x,y,z}}$ and so

$$-\sum_{x,y,z}r_{x,y,z}>\sum_{x,y,z}q_{x,y,z}\ln\frac{q_{x,y,z}}{t_{x,y,z}}.$$

 ☐

The dual problem of (EXP) is

$$\begin{array}{cc} \mathrm{maximize} & -\sum_{x,y}\lambda_{x,y}p_{x,y,*}-\sum_{x,z}\lambda_{x,z}p_{x,*,z} \end{array}$$

(9a) $$\begin{array}{ccc} \mathrm{subject}\;\mathrm{to}\; & \nu_{x,y,z}^{1}=-1 & \mathrm{for}\;\mathrm{all}\;(x,y,z)\in X\times Y\times Z \end{array}$$

(9b) $$\begin{array}{ccc} & -\mu_{x,y,z}+\nu_{x,y,z}^{2}=0 & \mathrm{for}\;\mathrm{all}\;(x,y,z)\in X\times Y\times Z \end{array}$$

(9c) $$\begin{array}{ccc} & -\mu_{*,y,z}-\lambda_{x,y}-\lambda_{x,z}+\nu_{x,y,z}^{3}=0 & \mathrm{for}\;\mathrm{all}\;(x,y,z)\in X\times Y\times Z \end{array}$$

(9d) $$\begin{array}{ccc} & \left(\nu_{x,y,z}^{1},\nu_{x,y,z}^{2},\nu_{x,y,z}^{3}\right)\geq_{K_{\exp}^{*}}0 & \mathrm{for}\;\mathrm{all}\;(x,y,z)\in X\times Y\times Z \end{array}$$

Using the definition of $K_{\exp}^{*}$ the system consisting of (9a)–(9d) is equivalent to

$$\lambda_{x,y}+\lambda_{x,z}+\mu_{*,y,z}+1+\ln\left(-\mu_{x,y,z}\right)\geq 0\;\;\mathrm{for}\;\mathrm{all}\;(x,y,z)\in X\times Y\times Z$$

and so the dual problem of (EXP) can be formulated as

(D-EXP) $$\begin{array}{ccc} \mathrm{maximize} & -\sum_{x,y}\lambda_{x,y}p_{x,y,*}-\sum_{x,z}\lambda_{x,z}p_{x,*,z}\\ \mathrm{subject}\;\mathrm{to}\; & \lambda_{x,y}+\lambda_{x,z}+\mu_{*,y,z}+1+\ln\left(-\mu_{x,y,z}\right)\geq 0 & \mathrm{for}\;\mathrm{all}\;(x,y,z)\in X\times Y\times Z \end{array}$$

**Proposition** **2.** *Strong duality holds for the primal-dual pair *(EXP)* and* (D-EXP).

**Proof.**  We assume that $p_{x,y,*},p_{x,*,z}>0.$ Consider the point $\tilde{s}$ with ${\tilde{s}}_{x,y,z}=\left({\tilde{r}}_{x,y,z},{\tilde{t}}_{x,y,z},{\tilde{q}}_{x,y,z}\right)$ such that

(10) $$\begin{array}{cc} {\tilde{r}}_{x,y,z} & :={\tilde{q}}_{x,y,z}\log\frac{{\tilde{p}}_{x,y,z}}{{\tilde{q}}_{x,y,z}}-100\\ {\tilde{t}}_{x,y,z} & :={\tilde{q}}_{*,y,z}\\ {\tilde{q}}_{x,y,z} & :=\frac{p_{x,y,*}\cdot p_{x,*,z}}{p_{x,*,*}}. \end{array}$$

$\tilde{s}$ is an interior point of (EXP). We refer to [14] for the proof. Hence, by Theorem 1, strong duality holds for the primal-dual pair (EXP) and (D-EXP). ☐

Weak and strong duality in their turn provide a measure for the quality of the returned solution, for more details see Section 3.4.

## 3. The BROJA_2PID Estimator

We implemented the exponential cone program (EXP) in Python and used a conic optimization solver to get the desired solution. Note that we are aware of only two conic optimization software toolboxes which allow solving Exponential Cone Programs, ECOS and SCS. The current version of Broja_2pid utilizes ECOS to solve the Exponential Cone Program (EXP). ECOS (we use the version from 8 November 2016) is a lightweight numerical software for solving Convex Cone programs [9].

This section describes the Broja_2pid package form the user’s perspective. We briefly explain how to install Broja_2pid. Then, we illustrate the framework of Broja_2pid and its functions. Further, we describe the input, tuning parameters, and output.

### 3.1. Installation

To install Broja_2pid, you need Python to be installed on your machine. Currently, you need to install ECOS, the Exponential Cone solver. To do that, you most likely pip3 install ecos. If there are troubles installing ECOS, we refer to its Github repository https://github.com/embotech/ecos-python. Finally, you need to gitclone the Github link of Broja_2pid and it is ready to be used.

### 3.2. Computing Bivariate PID

**Figure 2:**
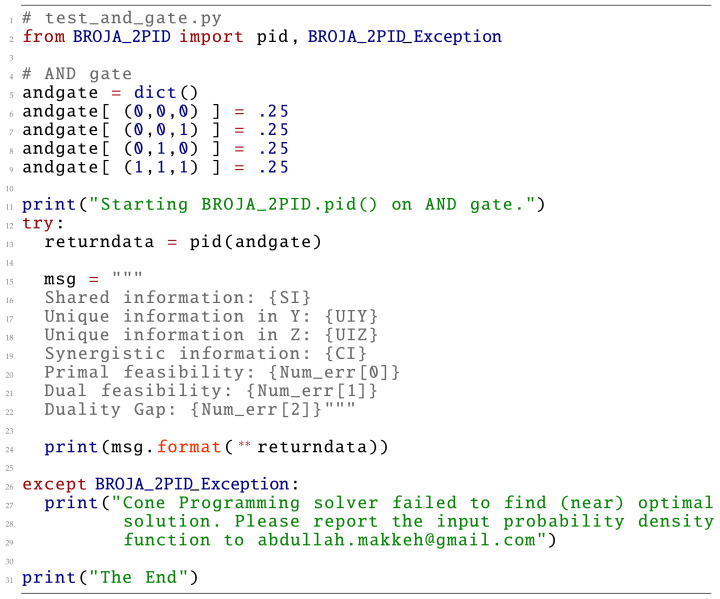
Computing the partial information decomposition of the And gate using Broja_2pid.

In this subsection, we will explain how Broja_2pid works. In Figure 2, we present a script as an example of using Broja_2pid package to compute the partial information decomposition of the And distribution, X=YANDZ where *Y* and *Z* are independent and uniformly distributed in {0,1}.

We will go through the example (Figure 2) to explain how Broja_2pid works. The main function in Broja_2pid package is pid(). It is a wrap up function which is used to compute the partial information decomposition. First, pid() prepares the “ingredients” of (EXP). Then, it calls the Cone Programming solver to find the optimal solution of (EXP). Finally, it receives from the Cone Programming solver the required solution to compute the decomposition.

The “ingredients” of (EXP) are the marginal and coupling equations, generalized inequalities, and the objective function. Thus, pid() needs to compute and store $p_{x,y,*}$ and $p_{x,*,z}$, the marginal distributions of (X,Y) and (X,Z). For this, pid() requires a distribution of X,Y, and *Z*. In Figure 2, the distribution comes from the And gate where X=YANDZ.

Distributions are stored as a Python dictionary data structure in which the random variable (x,y,z) is a *triplet* key and the probability at this point is the *value*. This provides an associate memory structure where the value of the random variable is a reference to the probability at that point. For example, the triplet (0,0,0) occurs with probability 1/4 and so on for the other triplets. Thus, And distribution is defined as a Python dictionary, andgate=dict() where andgate[ (0,0,0) ]=0.25 is assigning the key “(0,0,0)” a value “0.25” and so on.

Note that the user does not need to add the triplets with zero probability to the dictionary since pid() will always discard such triplets. In [5], the authors discussed in details how to handle the triplets with zero probability. The input of pid() is explained in details in the following subsection.

**Figure 3:**
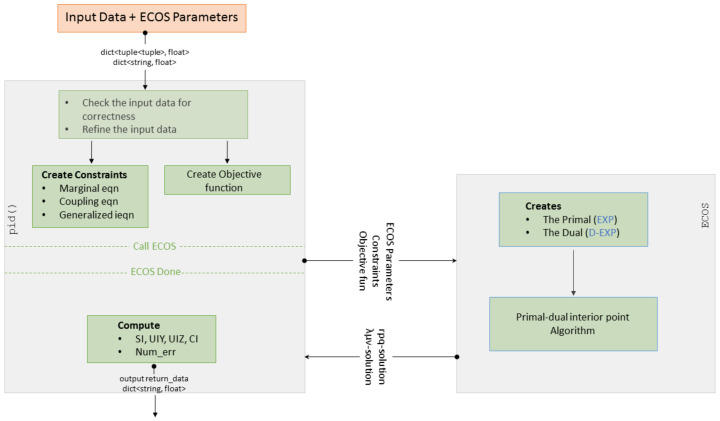
Broja_2pid workflow: (**Left**) the flow in pid(); and (**Right**) the flow in ECOS. The arrows with oval tail indicate passing of data, whereas the ones with line tail indicate time flow.

Now, we briefly describe how pid() proceeds to return the promised decomposition. pid() calls the Cone Programming solver and provides it with the “ingredients” of (EXP) as a part of the solver’s input. The solver finds the optimal solution of (EXP) and (D-EXP). When the solver halts, it returns the primal and dual solutions. Using the returned solutions, pid() computes the decomposition based on (1). The full process is explained in Figure 3.

Finally, pid() returns a Python dictionary, returndata containing the partial information decomposition and information about the quality of the Cone Programming solver’s solution. In Section 3.4, we give a detailed explanation on how to compute the quality of the solution and Table 3 contains a description of the keys and values of returndata.

For example, in the returned dictionary returndata for the And gate, returndata[’CI’] contains the quantity of synergistic information and returndata[’Num_err’][0] the maximum primal feasibility violation of (EXP).

Note that conic optimization solver is always supposed to return a solution. Thus, Broja_2pid will raise an exception, BROJA_2PID_Exception, when no solution is returned.

### 3.3. Input and Parameters

In Broja_2pid package, pid() is the function which the user needs to compute the partial information decomposition. The function pid() takes as input a Python dictionary.

The Python dictionary represents a probability distribution. This distribution computes the vectors $p_{x,y,*}$ and $p_{x,*,z}$ for the the marginal expressions in (EXP). A key of the Python dictionary is a *triplet* of (x,y,z) which is a possible outcome of the random variables X,Y, and *Z*. A value of the key (x,y,z) in the Python dictionary is a *number* which is the probability of X=x,Y=y, and Z=z.

The Cone Programming solver has to make sure while seeking the optimal solution of (EXP) that *w* and (η,θ) are feasible and (ideally) should halt when the duality gap is zero, i.e., *w* and (η,θ) are optimal. However, *w* and (η,θ) entries belong to R and computers represent real numbers up to floating precision. Thus, the Cone Programming solver considers a solution feasible when none of the constraints are violated, or optimal, duality gap is zero, up to a numerical precision (tolerance). The Cone Programming solver allows the user to modify the feasibility and optimality tolerances along with couple other parameters which are described in Table 1.

**Figure 4:**
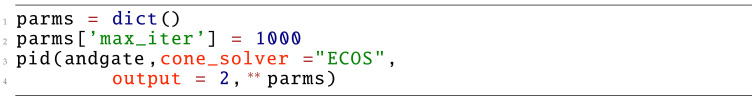
Tuning parameters.

To change the default Cone Programming solver parameters, the user should pass them to pid() as a dictionary. For example, in Figure 4, we change the maximum number of iterations which the solver can do. For this, we created a dictionary, parms=dict(). Then, we set a desired value, 1000, for the key ’max_iter’. Finally, we are required to pass parms to pid() as a dictionary, pid(andgate,^∗∗^parms). Note that, in the defined dictionary parms, the user only needs to define the keys for which the user wants to change the values.

The parameters output determines the printing mode of pid() and is an integer in {0,1,2}. This means that it allows the user to control what will be printed on the screen. Table 2 gives a detailed description of the printing mode.

Currently, we only use ECOS to solve the Exponential Cone Program but in the future we will add the SCS solver. Thus, the user should determine which solver to use in the computations. For exmple, setting cone_solver=“ECOS” will utilize ECOS in the computations.

### 3.4. Returned Data

The function pid() returns a Python dictionary called returndata. Table 3 describes the returned dictionary.

Let w,η, and θ be the lists returned by the Cone Programming solver where $w_{x,y,z}=\left[r_{x,y,z},t_{x,y,z},q_{x,y,z}\right],$ $\eta_{x,y,z}=\left[\lambda_{x,y},\lambda_{x,z},\mu_{x,y,z}\right],$ and $\theta_{x,y,z}=\left[\nu_{x,y,z}\right]$. Note that *w* is the primal solution and (η,θ) is the dual solution. The dictionary returndata gives the user access to the partial information decomposition, namely, shared, unique, and synergistic information. The partial information decomposition is computed using only the positive values of $q_{x,y,z}$. The value of the key ’Num_err’ is a triplet such that the primal feasibility violation is returndata[’Num_err’][0], the dual feasibility violation is returndata[’Num_err’][1], and returndata[’Num_err’][2] is the duality gap violation. In the following, we will explain how we compute the violations of primal and dual feasibility in addition to that of duality gap.

The primal feasibility of (EXP) is

(11) $$\begin{array}{c} q_{x,y,*}=p_{x,y,*}\\ q_{x,*,z}=p_{x,*,z}\\ q_{*,y,z}=t_{x,y,z}\\ \left(-r_{x,y,z},-t_{x,y,z},-q_{x,y,z}\right)\leq_{K_{\exp}}0 \end{array}$$

We check the violation of $q_{x,y,z}\geq 0$ which is required by $K_{\exp}$. Since all the non-positive $q_{x,y,z}$ are discarded when computing the decomposition, we check if the marginal equations are violated using only the positive $q_{x,y,z}$. The coupling equations are ignored since they are just assigning values to the $t_{x,y,z}$ variables. Thus, returndata[’Num_err’][0] (primal feasibility violation) is computed as follows,

$$\begin{array}{cc} q_{x,y,z}^{\prime } & =\left\{\begin{array}{cc} 0 & \mathrm{if}\;q_{x,y,z}\leq 0\\ q_{x,y,z} & \mathrm{otherwise} \end{array}\right.\\ \mathrm{returndata}[{}^{\prime }\mathrm{Num}\_{\mathrm{err}}^{\prime }]\left[0\right] & =\max_{x,y,z}\left(|q_{x,y,*}^{\prime }-p_{x,y,*}|,|q_{x,*,z}^{\prime }-p_{x,*,z}|,-q_{x,y,z}\right) \end{array}$$

The dual feasibility of (D-EXP) is

(12) $$\lambda_{x,y}+\lambda_{x,z}+\mu_{*,y,z}+1+\ln\left(-\mu_{x,y,z}\right)\geq 0$$

For dual feasibility violation, we check the non-negativity of (12). Thus, the error returndata[’Num_err’][1] is equal to

$$\min_{x,y,z}\left(\lambda_{x,y}+\lambda_{x,z}+\mu_{*,y,z}+1+\ln\left(-\mu_{x,y,z}\right),0\right)$$

When *w* is the optimal solution of (EXP), we have

$$-\sum_{x,y,z}r_{x,y,z}=\sum_{x,y,z}q_{x,y,z}\log\frac{q_{x,y,z}}{q_{*,y,z}}=-H\left(X|Y,Z\right).$$

The duality gap of (EXP) and (D-EXP) is

(13) $$-H\left(X|Y,Z\right)+\lambda^{T}b,$$

where

$$\lambda^{T}b=\sum_{x,y}\lambda_{x,y}p_{x,y,*}+\sum_{x,z}\lambda_{x,z}p_{x,*,z}.$$

Since weak duality implies $H\left(X|Y,Z\right)\leq \lambda^{T}b$, we are left to check the non-negativity of (13) to inspect the duality gap. Thus, returndata[’Num_err’][2] is given by,

$$\max(-H\left(X|Y,Z\right)+\lambda^{T}b,0)$$

## 4. Tests

In this section, we test the performance of Broja_2pid on three types of instances. We describe the instances that Broja_2pid is tested against, report the results, and finally compare the performance of other estimators on the same instances. The two estimators that we compare the performance of Broja_2pid to, which produce reasonable results and we are aware of, are computeUI and ibroja. The first two types of instances are used as primitive validation tests. However, the last type of instances is used to evaluate the accuracy and efficiency of Broja_2pid in computing the partial information decomposition. We used a computer server with Intel(R) Core(TM) i7-4790K CPU (4 cores) and 16GB of RAM to compute PID for the instances. All computations of Broja_2pid and computeUI were done using one core, whereas ibroja used multiple cores.

### 4.1. Paradigmatic Gates

The following set of instances have been studied extensively throughout the literature. The partial information decomposition of the set of instances is known [2]. Despite their simplicity, they acquire desired properties of shared or synergistic quantities.

#### 4.1.1. Data

The first type of instances is based on the “gates” (Rdn, Unq, Xor, And, RdnXor, RdnUnqXor, and XorAnd) described in Table 1 of [1]. Each gate is given as a function (x,y,z)=G(W) which maps a (random) input *W* to a triple (x,y,z). The inputs are sampled uniformly at random, whereas, in Table 1 of [1], the inputs are independent and identically distributed.

#### 4.1.2. Testing

All the gates are implemented as dictionaries and pid() is called successively with different printing modes to compute them. The latter is coded into the script file at the Github directory Testing/test_gates.py. The values of the partial information decomposition for all the gates distributions (when computed by pid()) were equal to the actual values up to precision error of order $10^{-9}$ and the slowest time of computations is less than a millisecond.

#### 4.1.3. Comparison with Other Estimators

Both estimators, computeUI and ibroja, produced values of the partial information decomposition for all the gate distributions equal to the actual values up to precision error of order $10^{-10}$ but the slowest time of computations is more than ten milliseconds for computeUI and 12 s for ibroja.

### 4.2. *Copy* Gate

The Copy gate requires a large number of variables and constraints—see below for details. Thus, we used it to test the memory efficiency of the Broja_2pid estimator. Since its decomposition is known, it also provides to some extent a validation for the correctness of the solution in large systems.

#### 4.2.1. Data

Copy gate is the mapping of (y,z) chosen uniformly at random to a triplet (x,y,z) where x=(y,z). The Copy distribution overall size scales as ${\left|Y\right|}^{2}\times {\left|Z\right|}^{2}$ where y,z∈Y×Z. Proposition 18 in [1] shows that the partial information decomposition of Copy gate is

$$\begin{array}{cc} \mathrm{CI}(X;Y,Z) & =0\\ \mathrm{SI}(X;Y,Z) & =\mathrm{MI}(Y;Z)\\ \mathrm{UI}(X;Y\backslash Z) & =H(Y|Z)\\ \mathrm{UI}(X;Z\backslash Y) & =H(Z|Y) \end{array}$$

Since *Y* and *Z* are independent random variables, then UI(X;Y\Z)=H(Y) and UI(X;Z\Y)=H(Z) and SI(Y;Z)=0.

#### 4.2.2. Testing

The Copy distributions is generated for different sizes of Y and Z where Y=[m] and Z=[n] for m,n∈N\{0}. Then, pid() is called to compute the partial information decomposition for each pair of m,n. Finally, the returndata dictionary is printed along with the running time of the Broja_2pid estimator and the deviations of returndata[’UIY’] and returndata[’UIZ’] from H(Y) and H(Z), respectively. The latter process is implemented in Testing/test_large_copy.py. The worst deviation was of percentage at most $10^{-8}$ for any m,n≤90.

#### 4.2.3. Comparison with Other Estimators

**Figure 5:**
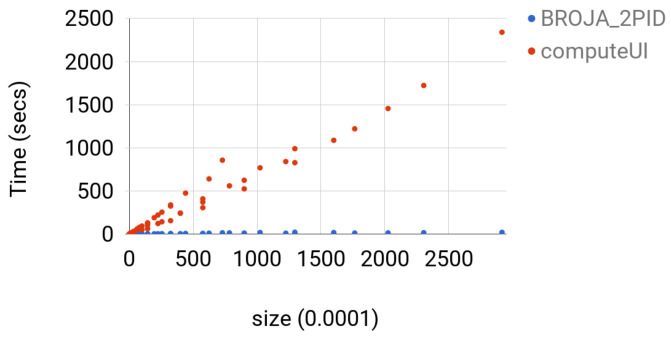
For each 10≤m≤60 and 10≤m≤90, the time for estimator computeUI and Broja_2pid for computing BROJA PID for the Copy gate with Y=n and Z=m is shown. The instances were arranged in increasing order with respect to the value of $m^{2}n^{2}$.

The ibroja estimator failed to give a solution to any instance since the machine was running out of memory. The computeUI estimator could solve instance of size less than or equal to 2.5exp7, but, for larger instances, the machine was running out of memory. computeUI was slower than Broja_2pid by at least a factor of 40 and at most factor of 113; see Figure 5 for comparison.

### 4.3. Random Probability Distributions

This is the main set of instances for which we test the efficiency of Broja_2pid estimator. It has three subsets of instance where each one is useful for an aspect of efficiency when the estimator is used against large systems. This set of instances had some hard distributions in the sense that the optimal solution lies on the boundary of feasible region of the problem (1).

#### 4.3.1. Data

The last type of instances are joint distributions of (X,Y,Z) sampled uniformly at random over the probability simplex. We have three different sets of the joint distributions depending on the size of X,Y, and Z.

(a) For Set 1, we fix |X|=|Y|=2 and vary |Z| in {2,3,…,14}. Then, for each size of *Z*, we sample uniformly at random 500 joint distribution of (X,Y,Z) over the probability simplex.
(b) For Set 2, we fix |X|=|Z|=2 and vary |Y| in {2,3,…,14}. Then, for each value of |Y|, we sample uniformly at random 500 joint distribution of (X,Y,Z) over the probability simplex.
(c) For Set 3, we fix |X|=|Y|=|Z|=s where s∈{8,9,…,18}. Then, for each *s*, we sample uniformly at random 500 joint distribution of (X,Y,Z) over the probability simplex.

Note that, in each set, instances are grouped according to the varying value, i.e., |Y|,|Z|, and *s*, respectively.

#### 4.3.2. Testing

The instances were generated using the Python script Testing/test_large_randompdf.py. The latter script takes as command-line arguments |X|,|Y|,|Z| and the number of joint distributions of (X,Y,Z) the user wants to sample from the probability simplex. For example, if the user wants to create the instance of Set 1 with |Z|=7, then the corresponding command-line is python3 test_large_randompdf.py 2 2 7 500. The script outputs the returndata along with the running time of Broja_2pid estimator for each distribution and finally it prints the empirical average over all the distributions of SI(X;Y,Z),UI(X;Y\Z),UI(X;Y\Z), CI(X;Y,Z), and of the running time of Broja_2pid estimator.

In the following, for each of the sets, we look at UI(X;Y\Z) to validate the solution, the returndata[’Num_err’] triplet to examine the quality of the solution, and the running time to analyze the efficiency of the estimator.

**Figure 6:**
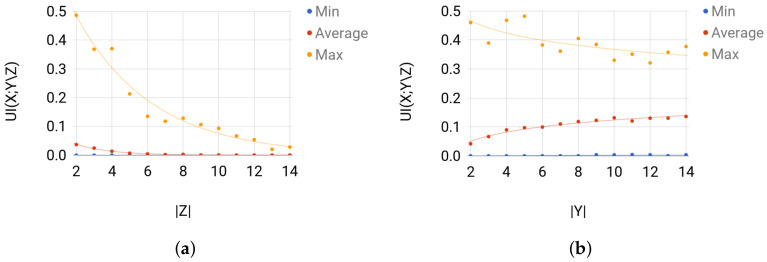
For each group of instances in Sets 1 and 2: (**a**) UI(X;Y\Z) of Set 1; and (**b**) UI(X;Y\Z) of Set 2 show the instance with the largest UI(X;Y\Z), the average value of UI(X;Y\Z) for the instances, and the instance with the smallest UI(X;Y\Z).

**Validation**. Sets 1 and 2 are mainly used to validate the solution of Broja_2pid. For Set 1, when |Z| is considerably larger than |Y|, the amount of unique information that *Y* has about *X* is more likely to be small for any sampled joint distribution. Thus, for Set 1, the average UI(X;Y\Z) is expected to decrease as the size of *Z* increases. For Set 2, UI(X;Y\Z) is expected to increase as the size of *Y* increases, i.e., when |Y| is considerably larger than |Z|. Broja_2pid shows such behavior of UI(X;Y\Z) on the instances of Sets 1 and 2 (see Figure 6).

**Figure 7:**
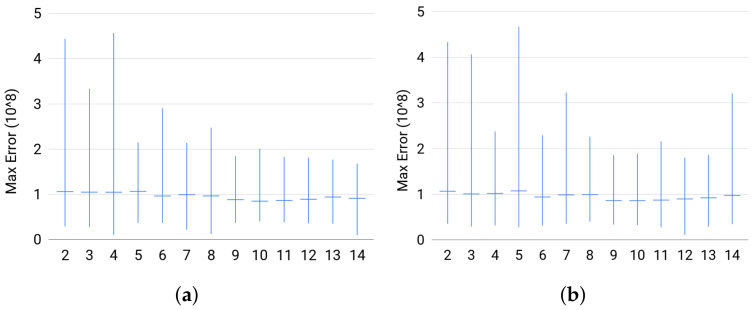

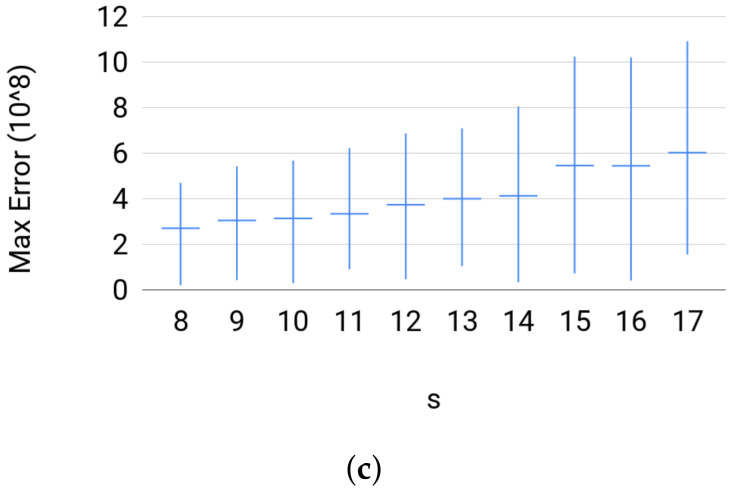
For each group of instances in Sets 1, 2, and 3: (**a**) maximum numerical error of Set 1; (**b**) maximum numerical error of Set 2; and (**c**) maximum numerical error of Set 3 show the instance with the largest *ϵ*, the average value of *ϵ* for the instances, and the instance with the smallest *ϵ*; where *ϵ* is the maximum numerical error.

**Quality**. The estimator did well on most of the instances. The percentage of solved instances to optimality was at least 99% for each size in any set of instances. In Figure 7, we plot the successfully solved instances against the maximum value of the numerical error triplet returndata[’Num_err’]. On the one hand, these plots show that, whenever an instance is solved successfully, the quality of the solution is good. On the other hand, we noticed that the duality gap, returndata[’Num_err’][2], was very large whenever the Cone Programming solver fails to find an optimal solution for an instance, i.e., the primal feasibility or dual feasibility or the duality gap is violated. In addition, even when Broja_2pid fails to solve an instance to optimality, it will return a solution. (Broja_2pidraise an exception if the conic optimization solver fails to return a solution.) Thus, these results reflect the reliability of the solution returned by Broja_2pid.

**Figure 8:**
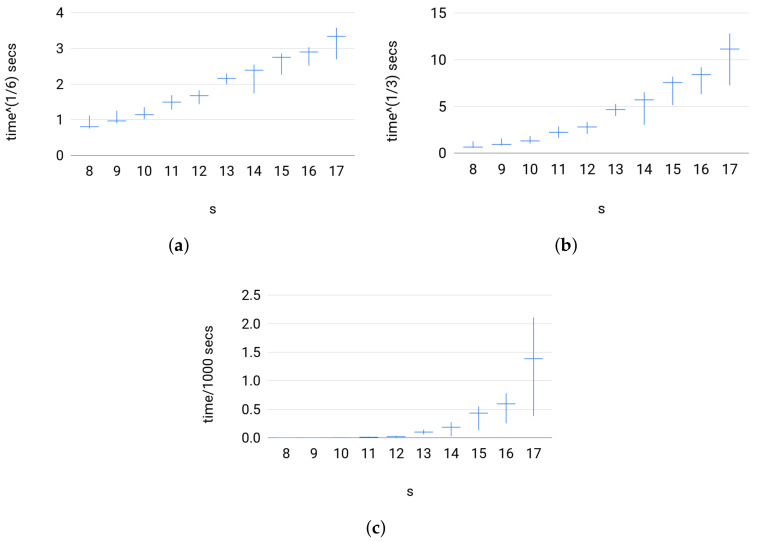
For each group of instances in Set 3: (**a**) $t^{1/6}$ versus *s*; (**b**) $t^{1/3}$ versus *s*; and (**c**) $t10^{3}$ versus *s* show the slowest instance, the average value of running times, and the fastest instance; where the running time of Broja_2pid, *t* (secs), is scaled to $t^{1/6},t^{1/3},$ and $t10^{3}$, respectively.

**Efficiency**. To test the efficiency of Broja_2pid in the sense of running time, we looked at Set 3 because Sets 1 and 2 are small scale systems.Set 3 has a large input size mimicking large scale systems. Testing Set 3 instances also reveals how the estimator empirically scales with the size of input. Figure 8 shows that the running time for Broja_2pid estimator against large instances was below 50 minutes. Furthermore, the estimator has a scaling of |X|×|Y|×|Z|, so, on Set 3, it scales as $N^{3}$ where *N* is the size of input for the sampled distributions such that |X|=|Y|=|Z|=N.

#### 4.3.3. Comparison with Other Estimators

We compare the Broja_2pid estimator with the computeUI estimator and ibroja against the instance of the same type of Set 3 for s={2,…,17}.

**Figure 9:**
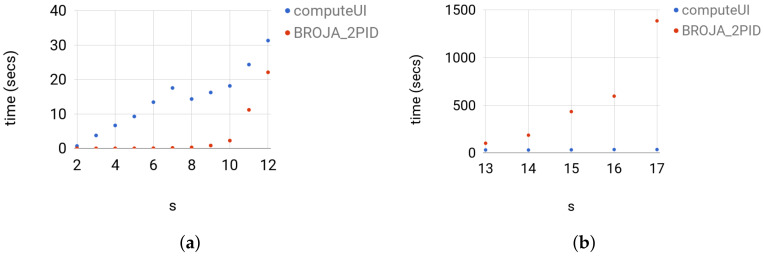
For each group of instances in Set 3: (**a**) *t* versus 2≤s≤12; and (**b**) *t* versus 13≤s≤17 show the running times of computeUI and Broja_2pid; where the running time *t* is in seconds.

**ComputeUI**: We ran computeUI with the default parameters, which are the -far from optimality $10^{-7}$, maximum outer iterations 1000, and maximum inner iteration 1000, for more details, see [7]. The estimator computeUI was slower than Broja_2pid on the instances of sizes |X|=|Y|=|Z|≤12 and faster on the larger instances. For |X|=|Y|=|Z|≤12, computeUI was slower than Broja_2pid by at least a factor of 1.4 and at most factor of 1330; see Figure 9 for the actual running times. For 13≤|X|=|Y|=|Z|≤17, computeUI was faster than Broja_2pid by at least a factor of 3.2 and at most factor of 39; see Figure 9 for the actual running times. The comparison shows that computeUI scales better than Broja_2pid on large instances, whereas on the regime |X|=||Y|=|Z|≤12, which is needed in practice, Broja_2pid scales better than computeUI.

Even though the optimality criterion of computeUI is $10^{-7}$, the solution of Broja_2pid was closer to the optima with a magnitude of $10^{-5}$ more than that of computeUI which concludes that Broja_2pid produces more enhanced solutions than those of computeUI. The test is implemented in Testing/test_from_file_broja_2pid_computeUI.py where the distributions in the folder randompdfs/ are the inputs.

**Ibroja**: The estimator ibroja is slower on any instances than Broja_2pid. For |X|=|Y|=|Z|≤7 ibroja was slower than Broja_2pid by at least a factor of 206 and at most factor of 6626. Note that the factor was increasing as |X|=|Y|=|Z| increases. We did not compute the instances of sizes |X|=|Y|=|Z|≥8 since ibroja started taking immensely long time to obtain the solutions for these instances.

The solution of Broja_2pid was closer to the optima with a magnitude of $10^{-3}$ for instances with s=… more than that of ibroja. Note that the results of this comparisons aligns with the claims imposed in [5] that first order methods are not suitable to tackle this problem. The test is implemented in Testing/test_from_file_dit.py where the distributions in the folder randompdfs/ are the inputs.

## 5. Cone Programming Model for Multivariate PID

Chicharro [8] introduced a multivariate PID measure using the so-called tree-base decompositions. The measure is similar to the bivariate BROJA PID measure yet it is not an extension of the BROJA PID measure. In this section, we show how to model some of the trivariate PID quantities using the exponential cone. Thus, with some modification, the Broja_2pid can be extended to compute some of the trivariate PID quantities. Note that, due to time constraint, we could not check whether the other trivariate PID quantities can be also fitted into the exponential cone.

Let S,X,Y,Z be random variables with finite range, where *S* is the target and X,Y,Z are the sources. Chicharro [8] defined the quantity of synergistic information that the sources X,Y,Z hold about the target *S* as:

(14) $$\mathrm{CI}\left(S;X_{1},X_{2},X_{3}\right)=\max\left(\mathrm{MI}\left(S;X,Y,Z\right)-\mathrm{MI}\left(S^{\prime };X^{\prime },Y^{\prime },Z^{\prime }\right)\right)$$

where the maximum extends over the triples of random variables $\left(S^{\prime },X^{\prime },Y^{\prime },Z^{\prime }\right)$ with the same 12,13,14-marginals as (S,X,Y,Z), i.e., $P\left(S=s,X=x\right)=P\left(S^{\prime }=s,X^{\prime }=x\right)$ for all s,x, $P\left(S=s,Y=y\right)=P\left(S^{\prime }=s,Y^{\prime }=y\right)$ for all s,y, and $P\left(S=s,Z=z\right)=P\left(S^{\prime }=s,Z^{\prime }=z\right)$ for all s,z. The objective function of (14), given the marginal conditions, is equal, up to a constant not depending on $S^{\prime },X^{\prime },Y^{\prime },Z^{\prime }$, to the conditional entropy $H(S^{\prime }|X^{\prime },Y^{\prime },Z^{\prime })$. Thus, we find that (14) is equivalent to the following Convex Program:

(15) $$\begin{array}{ccc} \mathrm{minimize} & \sum_{s,x,y,z}q_{s,x,y,z}\ln\frac{q_{s,x,y,z}}{q_{*,x,y,z}} & \mathrm{over}\;q\in R^{S\times X\times Y\times Z}\\ \mathrm{subject}\;\mathrm{to}\; & q_{s,x,*,*}=p_{s,x,*,*} & \mathrm{for}\;\mathrm{all}\;(s,x)\in S\times X\\ & q_{s,*,y,*}=p_{s,*,y,*} & \mathrm{for}\;\mathrm{all}\;(s,y)\in S\times Y\\ & q_{s,*,*,z}=p_{s,*,*,z} & \mathrm{for}\;\mathrm{all}\;(s,z)\in S\times Z\\ & q_{s,x,y,z}\geq 0 & \mathrm{for}\;\mathrm{all}\;(s,x,y,z)\in S\times X\times Y\times Z. \end{array}$$

Hence, the following Exponential Cone Program where the variables are $r,t,q\in R^{S\times X\times Y\times Z}$:

(16) $$\begin{array}{ccc} \mathrm{minimize} & -\sum_{s,x,y,z}r_{s,x,y,z}\\ \mathrm{subject}\;\mathrm{to}\; & q_{s,x,*,*}=p_{s,x,*,*} & \mathrm{for}\;\mathrm{all}\;(s,x)\in S\times X\\ & q_{s,*,y,*}=p_{s,*,y,*} & \mathrm{for}\;\mathrm{all}\;(s,y)\in S\times Y\\ & q_{s,*,*,z}=p_{s,*,*,z} & \mathrm{for}\;\mathrm{all}\;(s,z)\in S\times Z\\ & q_{*,x,y,z}-t_{s,x,y,z}=0 & \mathrm{for}\;\mathrm{all}\;(s,x,y,z)\in S\times X\times Y\times Z\\ & \left(-r_{s,x,y,z},-t_{s,x,y,z},-q_{s,x,y,z}\right)\leq_{K_{\exp}}0 & \mathrm{for}\;\mathrm{all}\;(s,x,y,z)\in S\times X\times Y\times Z. \end{array}$$

**Proposition** **3.** *The exponential cone program in *(16)* is equivalent to the Convex Program* (15).

**Proof.**  The proof follows in the same manner to that of Proposition 1. ☐

The dual problem of (16) can be formulated as

(17) $$\begin{array}{ccc} \mathrm{maximize} & -\sum_{s,x}\lambda_{s,x}p_{s,x,*,*}-\sum_{s,y}\lambda_{s,y}p_{s,*,y,*}-\sum_{s,y}\lambda_{s,z}p_{s,*,*,z}\\ \mathrm{subject}\;\mathrm{to}\; & \lambda_{s,x}+\lambda_{s,y}+\lambda_{s,z}+\mu_{*,x,y,z}+1+\ln\left(-\mu_{s,x,y,z}\right)\geq 0 & \mathrm{for}\;\mathrm{all}\;(s,x,y,z)\in S\times X\times Y\times Z. \end{array}$$

**Proposition** **4.** *Strong duality holds for the primal-dual pair *(16)* and* (17).

**Proof.**  The proof follows in the same manner to that of Proposition 2. ☐

Chicharro [8] defined the quantity of unique information that the sources *X* hold about the target *S* as:

(18) $$\mathrm{UI}\left(S;X\backslash Y,Z\right)=\min\mathrm{MI}\left(S^{\prime };X^{\prime },Y^{\prime },Z^{\prime }\right)-\min\mathrm{MI}\left(S^{\prime };Y^{\prime },Z^{\prime }\right)$$

where both minimums extend over the triples of random variables $\left(S^{\prime },X^{\prime },Y^{\prime },Z^{\prime }\right)$ with the same 12,13,14-marginals as $\left(S,X_{1},X_{2},X_{3}\right)$, i.e., $P\left(S=s,X=x\right)=P\left(S^{\prime }=s,X^{\prime }=x\right)$ for all s,x, $P\left(S=s,Y=y\right)=P\left(S^{\prime }=s,Y^{\prime }=y\right)$ for all s,y, and $P\left(S=s,Z=z\right)=P\left(S^{\prime }=s,Z^{\prime }=z\right)$ for all s,z. Analogously, he defines the unique information UI(S;Y\X,Z) and UI(S;Z\X,Y). Computing UI(S;X\Y,Z) consists of solving two optimization problems. The first problem in (18) can be formulated as (15) and the second problem can be formulated as follows:

(19) $$\begin{array}{ccc} \mathrm{minimize} & \sum_{s,y,z}q_{s,*,y,z}\ln\frac{q_{s,*,y,z}}{q_{*,*,y,z}} & \mathrm{over}\;q\in R^{S\times X\times Y\times Z}\\ \mathrm{subject}\;\mathrm{to}\; & q_{s,x,*,*}=p_{s,x,*,*} & \mathrm{for}\;\mathrm{all}\;(s,x)\in S\times X\\ & q_{s,*,y,*}=p_{s,*,y,*} & \mathrm{for}\;\mathrm{all}\;(s,y)\in S\times Y\\ & q_{s,*,*,z}=p_{s,*,*,z} & \mathrm{for}\;\mathrm{all}\;(s,z)\in S\times Z\\ & q_{s,x,y,z}\geq 0 & \mathrm{for}\;\mathrm{all}\;(s,x,y,z)\in S\times X\times Y\times Z. \end{array}$$

Hence, the following Exponential Cone Program where the variables are $r,t,q\in R^{S\times Y\times Z}$:

(20) $$\begin{array}{ccc} \mathrm{minimize} & -\sum_{s,y,z}r_{s,y,z}\\ \mathrm{subject}\;\mathrm{to}\; & q_{s,x,*,*}=p_{s,x,*,*} & \mathrm{for}\;\mathrm{all}\;(s,x)\in S\times X\\ & q_{s,*,y,*}=p_{s,*,y,*} & \mathrm{for}\;\mathrm{all}\;(s,y)\in S\times Y\\ & q_{s,*,*,z}=p_{s,*,*,z} & \mathrm{for}\;\mathrm{all}\;(s,z)\in S\times Z\\ & q_{*,*,y,z}-t_{s,y,z}=0 & \mathrm{for}\;\mathrm{all}\;(s,y,z)\in S\times Y\times Z\\ & \left(-r_{s,y,z},-t_{s,y,z},-q_{s,*,y,z}\right)\leq_{K_{\exp}}0 & \mathrm{for}\;\mathrm{all}\;(s,y,z)\in S\times Y\times Z. \end{array}$$

Similarly, we can prove the equivalence of (20) and (19) and that strong duality holds for (20) and its dual.

## 6. Outlook

We are aware of one other Cone Programming solver with support for the Exponential Cone, SCS [10]. We are currently working on adding the functionality to our software. When that is completed, giving the parameter cone_solver=“SCS” to the function pid() will make our software use the SCS-based model instead of the ECOS-based one. (The models themselves are in fact different: SCS requires us to start from the dual exponential cone program (D-EXP).) SCS employs parallelized first-order methods which can be run on GPUs, so we expect a considerable speedup for large-scale problem instances.

We may note that other information theoretical functions can also be fitted into the exponential cone. Thus, with some modification, the model can be used to solve other problems.

### Thanks

The authors would like to thank Patricia Wollstadt and Michael Wibral for their feedback on pre-production versions of our software. In addition, we thank Daniel Chicharro for fruitful discussions and pointing us to applications of our approach to estimate multivariate formulations of PID.

## Figures and Tables

**Table 1:** Parameters (tolerances) of ECOS. The parameter reltol is not recommended to be set higher. For more explanation, see https://github.com/embotech/ecos.

Parameter	Description	Recommended Value
feastol	primal/dual feasibility tolerance	$10^{-7}$
abstol	absolute tolerance on duality gap	$10^{-6}$
reltol	relative tolerance on duality gap	$10^{-6}$
feastol_inacc	primal/dual infeasibility *relaxed* tolerance	$10^{-3}$
abstol_inacc	absolute *relaxed* tolerance on duality gap	$10^{-4}$
reltol_inacc	*relaxed* relative duality gap	$10^{-4}$
max_iter	maximum number of iterations that “ECOS” does	100

**Table 2:** Description of the printing mode in pid().

Output	Description
0 (default)	pid() prints its output (python dictionary, see Section 3.4).
1	In addition to output=0, pid() prints a flags when it starts preparing (EXP).
2	and another flag when it calls the conic optimization solver.
	In addition to output=1, pid() prints the conic optimization solver’s output.
	(The conic optimization solver usually prints out the problem statistics and the status of optimization.)

**Table 3:** Description of returndata, the Python dictionary returned by pid().

Key	Value
’SI’	Shared information, SI(X;Y,Z).
	(All information quantities are returned in bits.)
’UIY’	Unique information of *Y*, UI(X;Y\Z).
’UIZ’	Unique information of *Z*, UI(X;Z\Y).
’CI’	Synergistic information, CI(X;Y,Z).
’Num_err’	information about the quality of the solution.
’Solver’	name of the solver used to optimize (CP).
	(In this version, we only use ECOS, but other solvers might be added in the future.)
